# The Effect of Workplace Mobbing on Positive and Negative Emotions: The Mediating Role of Psychological Resilience Among Nurses

**DOI:** 10.3390/healthcare13151915

**Published:** 2025-08-05

**Authors:** Aristotelis Koinis, Ioanna V. Papathanasiou, Ioannis Kouroutzis, Iokasti Papathanasiou, Dimitra Anagnostopoulou, Ioannis Androutsakos, Maria Papandreou, Ioulia Katsaiti, Nikolaos Tsioumas, Melpomeni Mourtziapi, Pavlos Sarafis, Maria Malliarou

**Affiliations:** 1 Department of Nursing, University of Thessaly, Gaiopolis Campus, Larissa-Trikala Ring-Road, 415 00 Larissa, Greece; psarafis@uth.gr (P.S.); malliarou@uth.gr (M.M.); 2 Laboratory of Education and Research of Trauma Care and Patient Safety, Department of Nursing, University of Thessaly, Gaiopolis Campus, Larissa-Trikala Ring-Road, 415 00 Larissa, Greece; ikouroutzis@uth.gr (I.K.); iokpapathan@uth.gr (I.P.); danagnostop@uth.gr (D.A.); 3 Psychometrics Laboratory, Research and the Clinical Simulation Laboratory (In-House Clinics), Department of Psychology, Aegean Omiros College, Panepistimiou 17, 105 64 Athens, Greece; i.androutsakos@aegeancollege.gr (I.A.); m.papandreou@aegeancollege.gr (M.P.); i.katsaiti@aegeancollege.gr (I.K.); n.tsioumas@aegeancollege.gr (N.T.); m.mourtziapi@aegeancollege.gr (M.M.)

**Keywords:** workplace mobbing, mental resilience, nurses, emotions, PANAS, WPVB, psychological well-being

## Abstract

**Background:** Workplace mobbing is a widespread phenomenon with serious psychological and emotional consequences on employees’ emotional well-being. Psychological resilience has been identified as a potential protective factor against such adverse outcomes. **Aim:** This study investigates the relationship between workplace mobbing and emotional well-being, as expressed through positive and negative affect, and examines the mediating role of psychological resilience in this association. **Methods:** Ninety nurses participated in this cross-sectional study. Data were collected using the Connor–Davidson Resilience Scale (CD-RISC), the Workplace Psychologically Violent Behaviors (WPVB) scale, and the Positive and Negative Affect Schedule (PANAS). Statistical analyses included correlation, multiple regression, and mediation using bootstrapped confidence intervals. **Results:** Resilience was strongly associated with positive affect (r = 0.74, *p* < 0.001) and inversely with negative affect (r = −0.46, *p* < 0.001). Mobbing was significantly related to increased negative affect (β = 0.12, *p* < 0.001) but not to positive affect. Resilience emerged as the strongest predictor of emotional outcomes and partially mediated the relationship between “Attack on professional role” and negative affect. **Conclusions:** Psychological resilience plays a key protective role in moderating the emotional impact of workplace mobbing. Enhancing resilience in healthcare professionals may mitigate the negative emotional effects of mobbing, although it does not fully buffer against all its consequences.

## 1. Introduction

Workplace mobbing, a form of systematic psychological harassment, represents a serious occupational stressor particularly prevalent in healthcare settings. It entails repeated negative behaviors directed toward an individual, leading to psychological distress and diminished job satisfaction [1]. The nursing profession, due to its hierarchical structure and emotionally demanding nature, is especially vulnerable to such dynamics [2]. Negative psychological consequences of mobbing include anxiety, depression, and burnout, while positive emotional states are often undermined [3]. Emotional well-being, conceptualized through the dual lens of positive and negative affect [4], has emerged as a critical indicator of both mental health and occupational performance. Understanding the factors influencing these affective dimensions is essential for promoting a healthier work environment. Psychological resilience, defined as the capacity to adapt successfully to adversity, has been proposed as a buffer against the negative impact of workplace stressors [5,6]. High resilience levels are associated with better coping strategies, lower emotional exhaustion, and improved affective functioning [7]. Empirical studies highlight the moderating or mediating roles of resilience in workplace stress contexts [8,9]. However, research specifically addressing the mediating role of resilience in the relationship between workplace mobbing and affect is scarce, particularly within nursing populations in European healthcare systems. This study aims to address this gap by exploring (1) the relationship between mobbing and affective states (positive and negative affect) and (2) the potential mediating effect of psychological resilience in these associations. Guided by the Conservation of Resources Theory (Hobfoll, 2011 [10]), which suggests that individuals strive to retain, protect, and build psychological resources to manage stress and prevent burnout, this study examines how workplace mobbing influences emotional well-being among emergency department nurses, and whether psychological resilience may act as a protective resource. By analyzing these relationships, this research aims to support the development of targeted interventions for improving the psychosocial climate in healthcare environments.

### 1.1. Literature Review

The phenomenon of mobbing, or workplace bullying, refers to repeated, systematic, and harmful behaviors that occur within the work environment with the intent of psychologically intimidating or isolating the employee [10]. The nursing profession—particularly staff in emergency departments (EDs)—appears to be especially vulnerable to such phenomena due to the nature of the work, high levels of stress, shift work, and interpersonal conflicts [11]. The work environment in emergency departments is particularly demanding and is often characterized by intense stress, resource shortages, unpredictable situations, and emotional exhaustion [12]. The emotional experience of nurses in this context is shaped by the interplay of these factors and manifests in both positive emotions (such as job satisfaction and a sense of contribution) and negative emotions (such as anger, anxiety, fear, and frustration) [13].

### 1.2. Emergency Department Nurses: A High-Risk Group

Emergency department (ED) nurses represent a highly vulnerable subgroup within healthcare due to the unpredictable and emotionally intense nature of their work [14]. High-pressure environments, overcrowding, staff shortages, and exposure to critical incidents contribute to elevated occupational stress and emotional fatigue. This constellation of stressors creates fertile ground for workplace mobbing to occur [15,16].

Several studies underline the consequences of this high-risk setting. Adriaenssens et al. [13] highlight the connection between insufficient organizational support and emotional exhaustion. Similarly, Johnson [17] found that ED nurses are particularly susceptible to both vertical and horizontal mobbing, which is closely linked to psychological distress. Wright and Khatri [18] added that ineffective leadership can intensify this problem, as nurses in these environments often feel powerless, a factor contributing to decreased resilience.

Addressing mobbing in EDs requires more than recognition—it demands structural interventions. Research supports the development of clear anti-bullying policies, systematic support structures, and resilience-enhancing training programs [6]. Interventions such as Mindfulness-Based Stress Reduction (MBSR) and cognitive–behavioral strategies have shown promise in improving coping mechanisms and emotional outcomes [18,19].

### 1.3. Mobbing in Healthcare Settings and Emergency Departments

Psychological violence in healthcare manifests in various forms, including verbal abuse, exclusion, and questioning of professional competence [20]. The phenomenon is not episodic but often manifests as a sustained pattern of aggression. A large multinational study revealed that over 30% of nurses reported being mobbed in the past year, with ED nurses experiencing the highest prevalence [21].

Einarsen et al. [1] emphasized the chronic nature of mobbing, describing it as a sequence of aggressive acts that can span weeks or months. The impact of such persistent behaviors on the psychological health of healthcare workers is profound, particularly in emotionally taxing environments like the ED.

### 1.4. Mobbing and Emotional Consequences

Mobbing is one of the strongest predictors of negative emotional states among healthcare professionals, especially in EDs [22]. Perpetrators often include both colleagues and supervisors, with behaviors ranging from professional devaluation to social isolation [16].

Studies consistently report heightened levels of emotional distress, including fear, anger, shame, and emotional exhaustion among those exposed to mobbing [23,24]. Simultaneously, positive affective states—such as enthusiasm and trust—tend to decline, contributing to emotional disengagement and reduced quality of care [25].

Quantitative findings support these patterns. For instance, Lin, W.-Q [26] and Soriano-Vázquez, I. [27] found that high exposure to mobbing significantly increased negative affect and symptoms of anxiety and depression. A survey of nursing staff in an Australian hospital showed that they reported exposure to workplace bullying and types of internal and external emotional abuse. In terms of antecedents, bullying was associated with high negative affectivity (NA) and low support from supervisor and colleagues. [28]. Karatuna et al. [29] argued that prolonged mobbing may even lead to emotional dysregulation. Other studies confirmed similar outcomes across different contexts; Greek Cypriot [30] and South Korean [31] nurses all reported strong correlations between workplace bullying and deteriorating emotional health. Environmental factors in EDs, such as overcrowding and critical case loads, appear to amplify these effects, exacerbating vulnerability to mobbing [32].

### 1.5. Emotional Experience in Emergency Departments

Emergency department (ED) nurses are routinely exposed to psychologically demanding situations, including patient deaths, severe injuries, and interactions with demanding patients and families, leading to the development of high levels of negative emotional responses [33]. The constant fluctuations in intensity, irregular shift schedules, and emotional involvement with patients further burden their mental well-being [34].

However, despite these adversities, many nurses report experiencing positive emotions such as satisfaction derived from providing care, a sense of purpose, and pride in their contribution to healthcare delivery [35]. This dual emotional experience renders the psychological state of ED nurses highly dynamic and particularly susceptible to external stressors such as mobbing.

Although mobbing intensifies negative emotional states, research indicates that it can coexist with positive feelings, such as professional fulfillment and empathy. A grounded theory study conducted in Taiwan identified key themes like “maintaining optimism” and preserving “compassion” as strategies for sustaining positive emotional states amid adversity [36]. This coexistence of positive and negative affective experiences is consistent with the dual-factor model of mental health, which posits that positive and negative emotions can operate independently [37]. Therefore, interventions that focus solely on reducing negative emotions may overlook opportunities to actively cultivate and enhance positive emotional experiences.

### 1.6. Psychological Resilience as a Mediator

Psychological resilience refers to an individual’s ability to adapt positively and “bounce back” from traumatic or stressful events while maintaining mental equilibrium [5]. Among emergency department (ED) nurses, resilience plays a crucial role in managing the negative emotional responses caused by mobbing and contributes to the enhancement of positive emotional experiences [38]. Research indicates that resilience functions either as a protective factor or as a mediator in the relationship between occupational stress and psychological well-being [39,40].

In a study by Hsieh et al. (2021), it was found that ED nurses with high levels of resilience experienced lower levels of negative affect and were less influenced by mobbing, regardless of the frequency of such incidents [41]. Similarly, the study by Arrogante, O. (2017) on ED nurses revealed that individuals with high psychological resilience exhibited reduced levels of stress and negative emotion, alongside increased levels of gratitude, optimism, and professional self-efficacy [42].

Hart et al. (2014) further demonstrated that resilience mediates the relationship between mobbing and mental well-being by reducing the impact of mobbing on negative emotions and enhancing resistance to psychological harassment [7]. Resilience has also been associated with the preservation of positive affect, even in adverse work environments, through mechanisms such as cognitive reappraisal, self-regulation, and social support [43].

In support of these findings, Yıldırım (2019) reported that resilience mitigated the impact of bullying on anxiety and depressive symptoms among nurses [44]. Likewise, Hegney et al. (2015) [45] found that resilience training significantly reduced emotional exhaustion and improved positive affect among nursing staff. These results suggest that resilience not only buffers the emotional burden of mobbing but also promotes psychological flexibility and optimism, thereby enhancing positive emotional outcomes [45].

A grounded theory study conducted in Taiwan with ED nurses exposed to workplace violence outlined a developmental process of resilience that included emotional expression, cognitive interpretation, and the adoption of coping strategies rooted in professional commitment [36]. Quantitative studies corroborate this pathway; Korean nurses who experienced workplace violence but exhibited high resilience reported improved job performance and lower levels of burnout [31].

Similarly, a survey of ED nurses in the United States found that resilience, along with moral distress, predicted workplace engagement. High resilience was associated with more favorable emotional outcomes and a lower intention to leave the profession [46]. A systematic review further confirmed that enhanced resilience reduces the risk of secondary traumatic stress and emotional exhaustion among ED nurses, with structured interventions such as Reflective Debriefing identified as effective support tools [47].

In qualitative research among Generation Z ED nurses in Shanghai, resilience was found to facilitate post-traumatic growth, enabling them to transform adversity into opportunities for personal and professional development [48]. Additionally, a 2024 thesis published in BMC Nursing examined factors contributing to ED nurses’ resilience in Iran. The study found that permanent employment, economic stability, and defined job roles significantly influenced resilience levels and reduced psychological strain [49].

Finally, an MDPI study concluded that resilience moderated the effects of workplace bullying on nursing outcomes. While resilience did not fully eliminate the adverse effects, it significantly strengthened nurses’ capacity to cope and maintain professional performance [50].

### 1.7. Scope and Aims of the Research

This study investigates the psychological impact of workplace bullying (mobbing) on nurses working in the emergency departments (EDs) of public hospitals within Greece’s 5th Regional Health Authority.

The primary aim of this research was to examine how exposure to mobbing affects nurses’ emotional states, focusing specifically on levels of positive and negative affect [4]. Additionally, the study explored the mediating role of psychological resilience, assessing whether resilience can buffer or alter the emotional consequences of workplace victimization [7,51]. By analyzing these relationships, the study aims to enhance understanding of the psychological mechanisms that may protect nurses from the adverse effects of mobbing and to support the development of targeted interventions in clinical settings [6].

## 2. Materials and Methods

### 2.1. Study Design

This study employed a cross-sectional, non-experimental, and descriptive design, appropriate for investigating relationships between psychological and workplace variables at a specific point in time. Specifically, the study aimed to explore the associations among workplace mobbing, positive and negative emotional states, and psychological resilience in nurses working in emergency departments (EDs).

Data collection was conducted using validated psychometric instruments, and statistical analyses appropriate for mediation modeling and inferential interpretation were applied. Each participant completed the measures once during a defined data collection period (October 2023 to March 2024), offering a snapshot of the emotional and workplace conditions experienced by ED nurses in four public hospitals.

A synchronic (single time-point) approach was used to collect data on three key constructs—workplace mobbing, psychological resilience, and the experience of positive and negative emotions—simultaneously. This design enabled the examination of prevalence, interrelationships, and group differences without inferring causation [52]. The absence of intervention or longitudinal follow-up positions this research firmly within the cross-sectional observational framework.

This methodological choice was influenced by the practical constraints of the high-intensity ED environment, where sustained or experimental intervention would be disruptive and ethically complex. The descriptive component of the design aimed to document the frequency and characteristics of workplace mobbing among ED nurses, while the analytical aspect examined how psychological resilience and emotional responses (positive and negative affect) were associated with mobbing experiences.

While cross-sectional designs do not permit causal inferences, they are well-suited to identifying patterns, correlations, and risk indicators, serving as a basis for future longitudinal or interventional research focused on preventing or mitigating moral harassment in healthcare settings. This design was therefore appropriate given the resource constraints and dynamic nature of emergency care environments, allowing for the investigation of potential psychological correlates of mobbing without disrupting clinical operations [52,53,54].

### 2.2. Place of Conduct

The research was conducted in the emergency departments of four public hospitals under the jurisdiction of the 5th Regional Health Authority (DYPE), which covers Thessaly and Central Greece.

Hospitals were selected based on the following explicit inclusion criteria:

- High volume of emergency admissions, ensuring exposure to high-stress clinical environments, a factor known to influence workplace behaviors and dynamics [55];
- Presence of full-time ED nursing staff, ensuring consistency in participants’ work settings and minimizing variability due to shift-based or temporary personnel [56];
- Institutional willingness to participate, including administrative approval and ethical clearance, in accordance with standard protocols for healthcare research [57];
- Variability in organizational structure and culture, allowing for a broader representation of workplace conditions, which is critical for understanding how organizational context influences mobbing and related psychosocial phenomena [58,59].

Although all hospitals function under the same regional authority, they differ in staffing models, institutional culture, and patient demographics—all factors that may influence the nature and perception of workplace mobbing. Including multiple institutions enhanced the ecological validity and generalizability of the study [60].

### 2.3. Study Sample

The study included 90 full-time nursing professionals, comprising both registered nurses (RNs) and nurse assistants, employed in the emergency departments (EDs) of four public general hospitals under the jurisdiction of the 5th Regional Health Authority in Thessaly, Central Greece. Participants were recruited from the General Hospitals of Larissa, Karditsa, Trikala, and Volos between February and March 2024.

The final sample was predominantly female (84.3%), with a mean age of 43.1 years (SD = 8.4). All participants worked in rotating shifts and were regularly exposed to high-acuity clinical situations characteristic of emergency care.

#### 2.3.1. Inclusion Criteria

Participants were eligible for inclusion if they met the following criteria:

- Were employed full-time in an ED at one of the participating hospitals;
- Were licensed nursing personnel, including registered nurses and nurse assistants [61];
- Had a minimum of six months’ continuous service in their current ED position (to ensure adequate exposure to the environment) [62];
- Had sufficient proficiency in Greek to understand and complete the questionnaire;
- Provided informed written consent to participate in the study.

#### 2.3.2. Exclusion Criteria

Participants were excluded if they met the following criteria:

- Were on temporary leave, extended absence, or probationary contracts at the time of data collection;
- Held administrative or exclusively supervisory roles with minimal direct patient contact;
- Had less than six months of experience in the ED setting;
- Declined or failed to provide informed consent;
- Had participated in a similar survey within the past year (to reduce survey fatigue and response bias) [63].

### 2.4. Study Instruments

Four structured questionnaires were used in this study to collect data from participants. These included three standardized psychometric instruments assessing workplace mobbing, emotional states, and psychological resilience, as well as a researcher-developed demographic questionnaire.

#### 2.4.1. Workplace Psychological Violence Behavior (WPVB) Questionnaire

The Workplace Psychologically Violent Behaviors (WPVB) instrument, originally developed by Yildirim and Yildirim [63], was employed to assess the frequency and nature of psychological violence experienced by healthcare professionals. This comprehensive self-report tool consists of 33 items, structured across four core domains that reflect different dimensions of psychological violence in the workplace.

Isolation from work (11 items): This subscale captures behaviors that distance the individual from work-related communication, participation, and inclusion. Attacks on professional role and performance (9 items): This dimension measures verbal or symbolic assaults targeting the worker’s professional competence and role. Aggressive behaviors toward the person (9 items): This includes personal hostility or threats directed at the individual rather than their professional identity. Direct negative acts (4 items): This subscale addresses overt, harmful actions such as shouting or public humiliation. Participants rated each item on a 6-point Likert scale ranging from 0 (“never”) to 5 (“always”), indicating the frequency with which they experienced each behavior in the workplace over a defined period. The WPVB scale has demonstrated excellent internal consistency, with an overall Cronbach’s alpha (α) coefficient of 0.95. The subscales also exhibit strong reliability, with alpha values ranging from 0.70 to 0.91, affirming the psychometric robustness of the tool across its domains. For the purposes of this study, the instrument was culturally adapted and linguistically validated for use in the Greek context, following standard procedures for translation and back-translation [64]. Formal permission for both the use and adaptation of the tool was obtained from the original authors and relevant institutional bodies [65]. For this study, the Greek translation of the (WPVB) was used.

#### 2.4.2. Positive and Negative Affect Schedule (PANAS)

The Positive and Negative Affect Schedule (PANAS) is a 20-item psychometric tool developed to assess the frequency of positive affect (PA) and negative affect (NA)—two key dimensions of emotional experience. It consists of two subscales (10 items each), with respondents rating how often they have experienced specific emotions (e.g., “excited” for PA, “upset” for NA) on a 5-point Likert scale ranging from 1 (“Very slightly or not at all”) to 5 (“Extremely”). Total scores for each subscale range from 10 to 50. When the positive emotion is at a low level, it is expected that the person is in a state of sadness and lethargy, while when the negative emotion is at a high level, negative consequences for the person are expected, such as anger, fear, nervousness, and dissatisfaction. The Positive and Negative Emotion Scale, when used with short-term instructions tends to be sensitive to mood swings, while when used with long-term instructions, it tends to show stability of mood characteristics.

In this study, both subscales demonstrated high internal consistency, with Cronbach’s α = 0.85 for both PA and NA. The PANAS is particularly suitable for capturing the short-term affective states of healthcare professionals working in high-stress environments such as emergency departments [4]. Its dimensional approach allows for nuanced analysis of how different emotional responses are shaped by external stressors like workplace mobbing. For this study, the Greek translation of the PANAS was used.

#### 2.4.3. Connor–Davidson Resilience Scale (CD-RISC)

The Connor–Davidson Resilience Scale (CD-RISC-25) is a widely used, psychometrically validated instrument designed to measure psychological resilience, defined as the capacity to cope effectively with adversity, stress, and traumatic events. Originally developed by Connor and Davidson [5], the full version of the scale contains 25 self-report items, each rated on a 5-point Likert scale, ranging from 0 to 4. The total score ranges from 0 to 100, with higher scores indicating greater levels of resilience.

The CD-RISC-25 assesses five core dimensions of resilience.

- Personal competence, high standards, and tenacity—reflecting self-efficacy, persistence, and confidence in one’s problem-solving abilities.
- Trust in one’s instincts and tolerance of negative affect—encompassing adaptive emotional processing and intuitive decision-making.
- Positive acceptance of change and secure relationships—indicating flexibility, adaptability, and the ability to maintain meaningful interpersonal connections.
- Sense of control—measuring one’s perceived influence over life circumstances.
- Spiritual influences—capturing the role of faith or existential beliefs as sources of strength.

The CD-RISC demonstrated excellent internal consistency in the present sample (Cronbach’s α = 0.93) and strong test–retest reliability, with an intraclass correlation coefficient (ICC) of 0.87, confirming its stability over time, consistent with previous research showing high reliability across clinical and non-clinical populations [1]. This scale was particularly relevant given the study’s focus on resilience as a mediator in the relationship between workplace mobbing and emotional outcomes.

For this study, the Greek translation of the CD-RISC-25 was used.

#### 2.4.4. Demographic Questionnaire

In addition, a demographic questionnaire was developed by the researchers to collect information on participants’ sociodemographic and occupational characteristics, including age, gender, marital status, educational level, years of professional experience, employment status, shift work, and the specific hospital unit of employment. This information was essential for describing the sample and for conducting subgroup analyses. All instruments used in the study were available in Greek, either through prior validated translations or translated following standard forward–backward translation procedures to ensure cultural and linguistic accuracy [66,67].

### 2.5. Data Collection Process—Research Ethics

Ethical approval for the study was obtained from the Scientific Committees of all participating hospitals, in accordance with national regulations governing biomedical research in clinical settings. Prior to data collection, the study’s purpose and procedures were explained to the head nurses of the emergency departments, and written institutional permissions were secured. The distribution of the questionnaires was conducted in person by the principal researcher, ensuring voluntary participation and anonymity. The study was approved as part of a postdoctoral research project, with official documentation granted by the respective hospital administrations as follows: University General Hospital of Larissa (Protocol No. 6641/8-2-2024), General Hospital of Karditsa (Protocol No. 5236/28-3-2024), General Hospital of Trikala (Decision No. 162, Protocol No. 5744/10/26-2-2024). The study was conducted in full compliance with the principles of research ethics and good clinical practice, including informed consent, confidentiality, and the right to withdraw without consequences. Participation was voluntary, anonymous, and confidential. Completion time was 15–30 min, and questionnaires were returned within 2–6 weeks. The ethical framework adhered to national legislation and international guidelines on conducting research involving human participants in healthcare settings [68].

Given the ethically sensitive nature of researching psychological stress and mobbing in nursing professionals—who can be considered a vulnerable population—the study incorporated specific safeguards to ensure participants’ protection and dignity. Participation was entirely voluntary, and informed consent was obtained in writing prior to data collection. All responses were anonymous and treated with strict confidentiality, preventing any risk of identification or professional repercussions.

Although the study did not include a direct psychological intervention, participants were explicitly informed—both in the written consent and verbally—that they could seek support from their hospital’s occupational health or psychological services should the process of participation evoke emotional discomfort. The research team was also trained to identify and respond appropriately to signs of distress, although no such incidents were recorded during data collection.

The broader aim of the study was to contribute to improved understanding and future policy interventions in workplace mental health, with the ultimate goal of reducing the psychological burden on emergency nurses. As such, the indirect benefit of participation lies in the potential for healthcare systems to use the findings to design and implement targeted strategies that promote emotional well-being and prevent workplace victimization. This approach was approved by the relevant institutional ethics committee and complies with national and international ethical guidelines for research involving human subjects.

### 2.6. Statistical Analysis

Quantitative variables were tested for normality using the Kolmogorov–Smirnov criterion. Only WPVB scales were not found to be normally distributed. Quantitative variables were expressed as mean values (Standard Deviation) and as median (interquartile range), while categorical and ordinal variables were expressed as absolute and relative frequencies. Pearson correlations coefficients (r) or Spearman correlations coefficients (rho) were used to explore the association of two continuous variables. The coefficient is considered very high when it is above 0.9, high when it is 0.7–0.9, moderate when it is 0.5–0.7, low when it is 0.3–0.5, and very low when it is below 0.3 [69]. Multiple linear regression analysis was used with dependent the PANAS subscales. Adjusted regression coefficients (β) with standard errors (SE) were computed from the results of the linear regression analyses.

For the investigation of the mediating role of resilience in the association between WPVB and PANAS scales, the SPSS PROCESS macro (model 4) was used following Hayes guidelines [69]. A 5000-sample bootstrap procedure was used to estimate bias-corrected 95% confidence intervals (CIs) to test the significance of indirect effect of the relationships. Mediation is presented when the indirect effect is significant, i.e., if confidence intervals do not contain zero. According to Hayes [70] and colleagues [9,40], this bootstrapping procedure overcomes the limitations of the approaches highlighted by Baron et al. [71] and Sobel [72], yielding results that are more accurate and less affected by sample size. Full mediation is presented when the direct effect is not significant, while partial mediation is presented when the direct effect is significant. Model diagnostics (such as multicollinearity, residuals, etc.) were checked appropriately. Also, various interaction terms of the independent variables were checked and reported when found significant.

It was calculated that with the sample of 90 participants, the study will have 90% power to conduct a linear regression model at a significance level of 0.05 and for effect sizes equal to 0.25 or greater.

Internal consistency reliability was determined by the calculation of Cronbach’s alpha coefficient. Scales with reliabilities equal to or greater than 0.70 were considered acceptable.

Scoring for each questionnaire followed standardized procedures as outlined in their respective manuals:

- Workplace psychological violence behaviors (WPVB) subscale scores were computed by summing the responses within each domain (item range: 0–5), yielding both domain-specific and total scores, with higher scores reflecting greater exposure to psychological violence in the workplace.
- The Connor–Davidson Resilience Scale (CD-RISC-25) total resilience score was derived by summing all 25 items (score range: 0–100), where higher scores indicate greater psychological resilience [5].
- The Positive and Negative Affect Schedule (PANAS) scores were calculated separately for the positive affect (PA) and negative affect (NA) subscales. Each subscale consists of 10 items rated on a 5-point Likert scale (1 = “very slightly or not at all” to 5 = “extremely”), with scores ranging from 10 to 50 per subscale. Higher PA scores reflect greater experience of positive emotions, while higher NA scores indicate more frequent negative emotional states [4].

Data completeness was verified before initiating statistical analyses. All participants completed the full set of instruments, including demographic information, with no missing values, ensuring data integrity and eliminating the need for imputation or case-wise exclusion.

All reported p values were two-tailed, with significance set at *p* < 0.05. Analyses were conducted using IBM SPSS Statistics, version 27.0, and Hayes’ PROCESS macro was used to test the mediation model (Model 4) [39].

**Figure 1:**
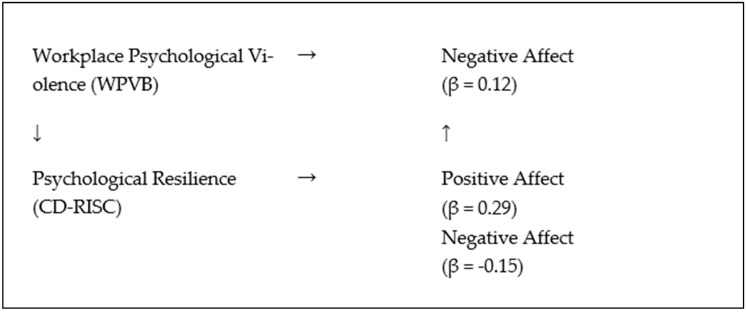
Conceptual mediation model illustrating the direct and indirect (mediated) relationships between workplace psychological violence (WPVB), psychological resilience (CD-RISC), and positive and negative affect (PA/NA). Note: Resilience partially mediated the relationship between WPVB (specifically ‘Attack on professional role’) and negative affect. WPVB was not significantly associated with positive affect.

To guide and visualize the analysis, a conceptual mediation model was developed and is presented in Figure 1. The elements in this model were as follows:

- Workplace psychological violence (WPVB) functions as the independent variable;
- Resilience (CD-RISC-25) serves as the mediating variable;
- Positive and Negative Affect (PANAS) are considered the outcome variables.

This model allowed for the investigation of both direct and indirect effects of workplace mobbing on emotional states through the buffering mechanism of resilience, reflecting the psychological dynamics experienced by nurses in emergency care settings.

Figure 1 represents the conceptual model used in the study, illustrating the direct and mediated relationships among workplace psychological violence (WPVB), psychological resilience (CD-RISC), and emotional outcomes (positive and negative affect).

## 3. Results

A total of 90 full-time nursing professionals participated in the study, yielding a response rate of 75%, based on the distribution of 120 self-administered questionnaires. All participants were actively employed in emergency departments across four public hospitals in the 5th Regional Health Authority of Greece. The sample was composed predominantly of women (n = 76; 84.3%), with a mean age of 43.1 years (SD = 8.4).

### 3.1. Specific Characteristics

Most participants (n = 64; 71.1%) held a Technological Educational Institute (TEI) degree, followed by MSc holders (n = 12; 13.3%), two-year college diplomas (n = 6; 6.7%), university (AEI) degrees (n = 5; 5.6%), and PhDs (n = 3; 3.3%). Marital status data indicated that 80.0% (n = 72) were married, 17.8% (n = 16) were unmarried, and 2.2% (n = 2) were divorced.

A total of 74.4% (n = 67) had children, most commonly two children (n = 38; 56.1%), followed by one child (n = 23; 34.8%) and three children (n = 6; 9.1%). The majority of participants (n = 76; 84.4%) reported living with others. In terms of professional status, nearly all respondents (n = 89; 98.9%) were registered nurses.

Regarding general health status, 52.2% (n = 47) reported being in good health. However, 21.1% (n = 19) reported chronic health conditions. Among those, the most frequently cited were arthritis or rheumatism (n = 6; 31.6%), heart problems (n = 3; 15.8%), hypertension (n = 3; 15.8%), and cancer (n = 3; 15.8%). Other reported conditions included leg problems (n = 2; 10.5%), diabetes (n = 1; 5.3%), and unspecified ailments (n = 2; 10.5%).

These demographic and health-related characteristics are summarized in Table 1 to provide relevant context for interpreting the psychological and occupational findings of the study.

Descriptive statistics for all major study variables are presented in Table 2. The mean score for the Connor–Davidson Resilience Scale (CD-RISC-25) was 66.38 (SD = 12.83), with scores ranging from 35 to 100. This reflects a moderate-to-high level of psychological resilience among participants. The positive affect subscale of PANAS showed a mean of 33.83 (SD = 5.35), while the negative affect subscale had a lower mean of 17.80 (SD = 6.02), indicating that participants reported more positive than negative emotional states overall. With regard to workplace victimization, the total workplace psychological violence behavior (WPVB) score had a mean of 17.87 (SD = 21.12), with wide variation observed across subscales. Specifically, mean scores for the WPVB subdomains were Attack on Personality: 4.78 (SD = 6.37); Attack on Professional Status: 5.74 (SD = 6.51); Individual’s Isolation from Work: 5.82 (SD = 7.61); and Direct Attack: 1.52 (SD = 3.38) All scales demonstrated high internal consistency, with Cronbach’s alpha coefficients ranging from 0.78 to 0.95. The CD-RISC-25 and the WPVB total score exhibited excellent reliability (α = 0.93 and α = 0.95, respectively), while the PANAS subscales and individual WPVB domains also met or exceeded the commonly accepted threshold of α > 0.70, indicating robust reliability across instruments.

As can be seen in Table 3, the levels of high resilience was significantly associated with more positive feelings (r = 0.74; *p* < 0.001) and less negative feelings (r = −0.46; *p* < 0.001). The negative feelings subscale was significantly and positively correlated with all scores regarding mobbing, while the positive feelings scale was not correlated at all with the WPVB scale. Psychological resilience, as measured by the CD-RISC, was strongly and positively associated with positive affect (r = 0.74, *p* < 0.001), indicating that individuals with higher resilience tend to experience greater positive emotions. Additionally, resilience showed a significant negative correlation with negative affect (r = −0.46, *p* < 0.001), suggesting that more resilient participants experience fewer negative emotional states. Regarding workplace victimization, negative affect was significantly and positively correlated with all WPVB subdomains, including attack on personality (ρ = 0.32, *p* = 0.002), attack on professional status (ρ = 0.27, *p* = 0.011), individual’s isolation from work (ρ = 0.26, *p* = 0.015), direct attack (ρ = 0.37, *p* < 0.001), and the total WPVB score (ρ = 0.33, *p* = 0.001). These findings highlight that individuals reporting more negative emotions also report higher exposure to workplace psychological violence. In contrast, positive affect was not significantly correlated with any WPVB subscale or the total score, indicating that positive emotional states appear unaffected by workplace victimization experiences. These results indicate that higher resilience scores were associated with greater positive affect and lower negative affect. Negative affect was also positively correlated with all domains of workplace psychological violence, whereas positive affect was not significantly correlated with any mobbing dimensions.

Table 4 presents the results of a multiple linear regression analysis conducted to identify predictors of positive emotional states, as measured by the positive affect subscale of the PANAS. Among all variables included in the model—demographic characteristics, health status, resilience (CD-RISC), and workplace psychological violence (WPVB)—only resilience emerged as a significant predictor. Via multiple linear regression analysis, with the positive feelings subscale as the dependent variable, it was found that only the score in the resilience scale was significantly associated with (β = 0.29; *p* < 0.001), indicating that resilience plays a key role in fostering positive emotional experiences. In contrast, the total WPVB score was not significantly related to positive affect (β = 0.04, *p* = 0.054), nor were any of the sociodemographic variables (all *p*-values > 0.17). These findings reinforce the central role of psychological resilience in emotional well-being, while suggesting that exposure to workplace victimization does not have a direct impact on the experience of positive emotions. Given that the total WPVB score was not significantly associated with positive affect in either the univariate (correlational) or multivariate analysis, no further mediation testing was warranted. Overall, the results underscore resilience as a crucial protective factor that enhances positive affect regardless of workplace stressors or individual background characteristics.

Table 5 presents the results of the multiple linear regression analysis with the negative affect subscale of the PANAS as the dependent variable. Several predictors emerged as statistically significant. Specifically, participants with a higher educational level reported significantly fewer negative feelings (β = −1.38, *p* = 0.031), suggesting that educational attainment may serve as a protective factor against negative emotional experiences. In contrast, participants who reported having a health problem exhibited significantly higher levels of negative affect (β = 3.49, *p* = 0.011), indicating the adverse emotional consequences of physical health issues. Psychological resilience was also a significant predictor, with higher resilience scores associated with lower levels of negative affect (β = −0.15, *p* = 0.001). Additionally, the total WPVB score was positively and significantly associated with negative affect (β = 0.12, *p* < 0.001), supporting the link between workplace victimization and negative emotional states. Further analyses explored the impact of individual WPVB subdomains. When entered into the regression model in turn (due to intercorrelations; Appendix A Table A1), the subscales “Attack on personality” (β = 0.41, *p* < 0.001), “Attack on professional status” (β = 0.32, *p* = 0.001), and “Individual’s isolation from work” (β = 0.31, *p* < 0.001) were all significantly associated with increased negative feelings. The “Direct attack” subscale did not reach statistical significance (β = 0.28, *p* = 0.137), possibly reflecting lower frequency or impact. Mediation analyses were conducted to assess whether resilience mediated the relationship between workplace victimization and negative affect. Resilience did not significantly mediate the association between the total WPVB score and negative affect, as the indirect effect was not statistically significant [indirect effect (95% CI) = 0.029 (−0.001, 0.080) and direct effect (95% CI) = 0.121 (0.064, 0.178)]. Similarly, no significant mediation effects were observed for the “Attack on personality” [indirect effect (95% CI) = 0.088 (−0.010, 0.214) and direct effect (95% CI) = 0.409 (0.218, 0.601)] or “Individual’s isolation from work” [indirect effect (95% CI) = 0.077 (−0.008, 0.237) and direct effect (95% CI) = 0.310 (0.157, 0.463)] subdomains. However, resilience was found to significantly and partially mediate the relationship between “Attack on professional status” and negative affect, as indicated by a significant indirect effect [indirect effect (95% CI) = 0.098 (0.008, 0.246)] and a remaining significant direct effect [direct effect (95% CI) = 0.319 (0.136, 0.502)]. This suggests that while professional attacks in the workplace have a direct negative impact on emotional well-being, resilience may buffer some of this effect.

### 3.2. Table 6—Mediation Analysis: Resilience as a Mediator

To examine whether psychological resilience mediated the relationship between workplace victimization and negative emotional outcomes, a series of mediation analyses were conducted. While resilience was hypothesized to buffer the emotional effects of workplace psychological violence, results showed that this mediation was not supported for most WPVB domains. However, a significant partial mediation effect was found for the association between “Attack on professional status” and negative affect, suggesting that resilience may partially mitigate emotional distress related to professional undermining.

To explore the potential psychological buffering role of resilience, a series of mediation analyses were conducted, with resilience score (CD-RISC) tested as a mediator in the association between workplace psychological violence and negative emotional states (negative affect). Specifically, indirect and direct effects were estimated using bias-corrected bootstrapping with 95% confidence intervals.

Results indicated that resilience did not significantly mediate the relationship between the total WPVB score and negative affect, as the 95% confidence interval for the indirect effect included zero [indirect effect = 0.029; 95% CI: −0.001, 0.080]. Similarly, no significant mediation was found for the WPVB subscales Attack on personality or Individual’s isolation from work. In these cases, although the direct effects on negative affect remained statistically significant, the indirect pathways through resilience were non-significant.

However, a significant partial mediation effect was observed for the association between “Attack on professional status” and negative affect. The indirect effect through resilience was statistically significant [indirect effect = 0.098; 95% CI: 0.008, 0.246] and the direct effect remained significant [direct effect = 0.319; 95% CI: 0.136, 0.502], indicating that resilience partially explained—but did not fully account for—the emotional impact of professional victimization.

These findings suggest that while resilience plays a protective role in emotional regulation, its mediating function may be context-specific, offering partial buffering particularly in cases of professional status undermining.

**Table 6:** Mediation analysis: resilience as mediator of the relationship between WPVB and negative affect.

Predictor Variable	Indirect Effect (Via Resilience)	95% CI (Indirect)	Direct Effect	95% CI (Direct)	Mediation?
Total WPVB Score	0.029	−0.001 to 0.080	0.121	0.064 to 0.178	✗ Not significant
Attack on Personality	0.088	−0.010 to 0.214	0.409	0.218 to 0.601	✗ Not significant
Attack on Professional Status	0.098	0.008 to 0.246	0.319	0.136 to 0.502	√ Partial mediation
Individual’s Isolation from Work	0.077	−0.008 to 0.237	0.310	0.157 to 0.463	✗ Not significant

Note: mediation effects were tested using bootstrapped confidence intervals; mediation was considered significant if the 95% CI for the indirect effect did not include zero.

## 4. Discussion

This study investigates the psychological impact of workplace bullying (mobbing) on nurses working in the emergency departments (EDs) of public hospitals within Greece’s 5th Regional Health Authority. The primary aim was to examine how exposure to mobbing affects nurses’ emotional states, focusing specifically on levels of positive and negative affect [73,74]. Additionally, the study explored the mediating role of psychological resilience, assessing whether resilience can buffer or alter the emotional consequences of workplace victimization [22,23]. By analyzing these relationships, this research seeks to enhance understanding of the psychological mechanisms that may protect nurses from the adverse effects of mobbing and to support the development of targeted interventions in clinical settings [75]. Collectively, these results emphasize the protective role of resilience in fostering positive emotions and mitigating negative affect, as well as the close link between negative emotions and experiences of workplace mobbing.

### 4.1. Key Findings of the Study—Summary

This study highlighted five key findings that are consistent with the existing international literature and are of particular importance for the mental health of nurses working in emergency departments (EDs).

Firstly, psychological resilience proved to be a significant protective factor, as it was positively associated with positive affect (r = 0.74, β = 0.29, *p* < 0.001) and negatively associated with negative affect (*p* = 0.001), demonstrating its role in enhancing mental well-being [43,76]. As described by Tugade and Fredrickson, individuals with high resilience utilize positive emotions to recover from stress and effectively manage critical situations [43].

Secondly, workplace psychological violence (mobbing) was positively associated with negative affect. Nurses exposed to professional identity attacks and social exclusion exhibited higher levels of anxiety, anger, and sadness—findings consistent with previous studies on the psychological burden of workplace aggression [74,77]. Mobbing is recognized as a significant risk factor for professional burnout and mental health problems in healthcare settings [22,23].

Thirdly, it was observed that resilience partially mediates the relationship between attacks on professional identity and negative affect. This means that while mobbing leads to negative emotions, the presence of psychological resources such as resilience can reduce the intensity of this impact [75,76]. This finding supports the Conservation of Resources Theory, which posits that individuals strive to preserve psychological reserves that protect them from exhaustion in stressful situations [78].

Fourthly, positive affect is associated with increased job satisfaction and improved quality of care. Nurses with higher levels of positive mood display greater enthusiasm, creativity, and ability to manage clinical pressure, positively affecting their relationships with patients [79,80].

Finally, the finding of partial mediation by resilience highlights that individual interventions alone are not sufficient. A combination of psychoeducational interventions (such as MBSR or CBT programs) and organizational strategies to prevent mobbing is required to holistically protect nurses’ mental health [7,81].

### 4.2. Detailed Discussion of the Research Findings

The following section provides a detailed discussion of the research findings, interpreting the results in the context of existing literature and theoretical frameworks. Each major outcome is analyzed in terms of its implications for clinical practice, organizational policy, and future research in the field of emergency nursing and workplace mental health.

### 4.3. Resilience as a Protective Factor

Our results demonstrated a strong positive correlation between resilience and positive affect (r = 0.74, β = 0.29, *p* < 0.001), alongside a moderate negative correlation with negative affect (r = –0.46, β = –0.15, *p* = 0.001). These findings align with prior studies emphasizing resilience as a vital psychological resource in high-stress healthcare settings. Tugade and Fredrickson originally proposed that resilient individuals employ positive emotions to mitigate the impact of stress and recover more effectively, which supports our observation that higher resilience corresponds to greater positive affect [43]. Similarly, in their meta-analysis, Kunzler et al. confirmed that resilience reduces vulnerability to burnout and psychological distress among healthcare professionals [75]. Liao, L. et al. further showed that resilience interventions improve emotional well-being in nurses exposed to workplace violence, reinforcing the protective role of resilience in emergency nursing contexts [80].

### 4.4. Impact of Workplace Psychological Violence on Emotional Health

Significant positive correlations were found between workplace psychological violence subdomains—including attacks on personality (ρ = 0.32, *p* = 0.002), direct threats (ρ = 0.37, *p* < 0.001), and isolation (ρ = 0.33, *p* = 0.003)—and negative affect. These results confirm the detrimental psychological impact of workplace violence, consistent with research highlighting bullying and verbal abuse as contributors to increased stress, anxiety, and emotional exhaustion among nurses [81]. Javaheri et al. also reported that workplace bullying significantly predicts negative emotional states in emergency department nurses, supporting our findings [82]. The observed mediation effect of resilience in attenuating the relationship between attacks on professional status and negative affect suggests that resilience may selectively buffer certain types of workplace violence, as indicated by Liao, L. et al., & Edwards et al., who noted that the effectiveness of resilience as a mediator may vary depending on violence severity and type [80,83].

### 4.5. Sociodemographic and Health Factors Influencing Emotional Affect

Lower educational attainment and existing health problems emerged as significant predictors of heightened negative affect (β = –1.38, *p* = 0.031 and β = 3.49, *p* = 0.011, respectively). These findings echo previous studies which show that nurses with chronic health conditions often report increased emotional distress due to the compounded burden of physical and occupational stressors [84,85]. Additionally, higher education is linked with better coping skills and emotional regulation capabilities, possibly explaining its inverse relationship with negative affect [86]. This emphasizes the importance of targeted support and professional development opportunities for nurses with lower educational levels to enhance their psychological resilience and emotional well-being.

### 4.6. Implications for Practice

The combined influence of workplace violence and individual resilience on emotional outcomes suggests that multifaceted interventions are necessary. Resilience training programs, such as those modeled by Liao, L. et al., have demonstrated efficacy in improving nurses’ psychological well-being and could be adopted more widely [80]. Concurrently, organizational policies aimed at minimizing workplace violence and fostering a psychosocially safe environment are crucial, consistent with Bailey, T. et al.’s findings that a positive psychosocial safety climate reduces psychological strain [85]. Leadership commitment, effective reporting systems, and staff education can mitigate workplace violence and its emotional consequences [87].

### 4.7. Implications and Strengths

This study offers valuable insights into the critical role of psychological resilience and workplace psychological violence on the emotional well-being of emergency nurses. By identifying resilience as a key protective factor that enhances positive affect and reduces negative emotional states, our findings support targeted interventions to build resilience in emergency care settings [88]. Such interventions may include mindfulness-based programs, peer support initiatives, and resilience training, which have been shown to help nurses better manage occupational stressors [7,89,90].

Furthermore, the study highlights the urgent need to address workplace psychological violence, particularly subtle forms such as attacks on professional status and social exclusion, which significantly harm nurses’ mental health [90,91]. The adoption of organizational policies and leadership commitment to foster a psychosocially safe work environment is essential to reduce these stressors [87].

A notable strength of this research is the use of reliable and validated measurement tools with strong internal consistency, enhancing the credibility of the results. The application of mediation analysis using bootstrap techniques further strengthens the validity of our conclusions, especially given the sample size [40,70]. Although limited by its cross-sectional design, the study lays groundwork for future longitudinal and interventional research aimed at improving mental health and working conditions of emergency nurses.

### 4.8. Limitations and Future Directions

It should be noted that multiple mediation models were tested in the current study, raising the possibility of Type I error due to multiple comparisons. Although one significant partial mediation was found (in the association between attack on professional status and negative affect), the remaining models did not yield statistically significant indirect effects. Therefore, the mediation results must be interpreted with caution, and future studies with larger sample sizes and adjusted statistical controls (e.g., Bonferroni correction or false discovery rate methods) are warranted to confirm these findings.

A convenience sampling method was used due to the unpredictable and high-pressure nature of emergency care environments, which complicates probability-based sampling strategies in clinical settings [57,61]. Despite this limitation, the sample represents a diverse cross-section of frontline emergency nursing staff across multiple institutions. The cross-sectional design limits causal inference and temporal understanding of the relationships studied. Furthermore, self-reported data might be influenced by response bias. The sample, though focused on Greek public hospitals, limits generalizability to other cultural or healthcare settings. Longitudinal studies are warranted to explore resilience dynamics over time and to assess the long-term impact of violence exposure. Future research should also investigate organizational and leadership variables as moderators or mediators in these relationships.

Additionally, the study exclusively involved nurses employed in public hospitals within Greece’s 5th Regional Health Authority, which may limit the generalizability of the findings to other healthcare settings. The structure, demands, and psychosocial environment of public sector emergency departments in Greece—characterized by understaffing, high patient flow, and rigid hierarchies—may differ significantly from those in private hospitals or primary care units. Consequently, the patterns of workplace mobbing, emotional response, and resilience observed in this context may not fully reflect the experiences of nurses working in other healthcare environments.

Future research should aim to include a more diverse and representative sample, encompassing nurses from both public and private sectors, as well as from urban and rural healthcare units, in order to explore potential contextual differences and enhance external validity. Such broader sampling would allow for a more nuanced understanding of how workplace dynamics and cultural or institutional variables influence psychological well-being in the nursing profession.

## 5. Conclusions

This study highlights psychological resilience as a vital protective factor for emergency department nurses, playing a crucial role in fostering positive affect and mitigating the negative emotional impacts of workplace psychological violence, particularly mobbing. The strong positive relationship between resilience and positive emotional states, alongside its partial mediation in buffering the adverse effects of attacks on professional identity, underscores its significance in sustaining mental well-being under the high-pressure conditions typical of emergency settings. Workplace mobbing was confirmed as a substantial risk factor contributing to heightened negative emotions such as anxiety, anger, and sadness, which threaten nurses’ psychological health and increase the risk of burnout. Conversely, positive affect was identified as a key driver of job satisfaction and improved patient care quality, demonstrating the broader organizational benefits of resilience cultivation. These findings emphasize that effective interventions must be multifaceted, combining individual resilience-building strategies—such as specialized training and peer support—with systemic organizational policies aimed at preventing workplace mobbing and fostering a psychologically safe and respectful environment. Such comprehensive approaches are essential to enhance nurses’ mental health, improve staff retention, and ultimately elevate the quality of emergency care.

Future longitudinal and experimental research is warranted to further elucidate causal mechanisms and evaluate the effectiveness of resilience-enhancement and anti-mobbing interventions across diverse healthcare contexts. Overall, this study contributes important insights into the complex interplay between individual psychological resources and workplace dynamics, advocating for holistic mental health promotion within nursing practice.

## Figures and Tables

**Table 1:** Sample characteristics (n = 90).

Variable	Category	n	%
Gender	Males	15	15.7
	Females	75	84.3
Educational level	2-year college	6	6.7
	Technological University	64	71.1
	University	5	5.6
	MSc	12	13.3
	PhD	3	3.3
Family status	Unmarried	16	17.8
	Married	72	80.0
	Divorced	2	2.2
Have children	No	23	25.6
	Yes	67	74.4
Number of children	1	23	34.8
	2	37	56.1
	3	6	9.1
Living	Alone	14	15.6
	With others	76	84.4
Professional	Nurse	89	98.9
	Assistant nurse	1	1.1
Educational level of nurses	Secondary education	6	6.7
	Technological university	74	82.2
	University	10	11.1
Location	VOLOS	19	21.3
	KARDITSA	25	28.1
	LARISSA	21	23.6
	TRIKALA	24	27.0
Health status	Very poor	5	5.6
	Poor	5	5.6
	Not poor. nor good	10	11.1
	Good	47	52.2
	Very good	23	25.6
Health problems		19	21.1
If yes, define	Heart problems	3	15.8
	Arthritis or rheumatism	6	31.6
	Emphysema or chronic bronchitis	0	0.0
	Cataract	0	0.0
	Bone fracture or crack	0	0.0
	Leg problems	2	10.5
	Parkinson’s disease	0	0.0
	Hypertension	3	15.8
	Cancer	3	15.8
	Diabetes	1	5.3
	Stroke	0	0.0
	Chronic mental health problems	0	0.0
	Rectal bleeding	0	0.0
	Other	2	10.5
		Mean	SD
Age (years)		43.1	8.4

**Table 2:** Descriptive measures for CD-RISC, WPVB, and PANAS scales.

	Minimum	Maximum	Mean (SD)	Median (IQR)	Cronbach’s Alpha
Resilience score (CD-RISC)	35.00	100.00	66.38 (12.83)	66.5 (58–74)	0.93
Positive feelings subscale (PANAS)	13.00	46.00	33.83 (5.35)	34.5 (30–37)	0.85
Negative feelings subscale (PANAS)	10.00	38.00	17.8 (6.02)	17 (13–21)	0.85
Attack on personality	0.00	29.00	4.78 (6.37)	2 (0–6)	0.87
Attack on professional	0.00	28.00	5.74 (6.51)	3 (1–8)	0.86
Individual’s isolation from work	0.00	45.00	5.82 (7.61)	4 (1–7)	0.89
Direct attack	0.00	19.00	1.52 (3.38)	0 (0–2)	0.78
Total WPVB score	0.00	110.00	17.87 (21.12)	9 (5–23)	0.95

**Table 3:** Correlation coefficients of PANAS subscales with CD-RISC and WPVB scales.

		Positive Feelings Subscale (PANAS)	Negative Feelings Subscale (PANAS)
Resilience score (CD-RISC)	r	0.74	−0.46
	*p*	<0.001	<0.001
Attack on personality	rho	−0.02	0.32
	*p*	0.884	0.002
Attack on professional	rho	−0.12	0.27
	*p*	0.258	0.011
Individual’s isolation from work	rho	−0.17	0.26
	*p*	0.108	0.015
Direct attack	rho	−0.08	0.37
	*p*	0.430	<0.001
Total WPVB score	rho	−0.13	0.33
	*p*	0.234	0.001

Note. Pearson’s correlation coefficients (r) and Spearman’s correlation coefficients (rho) are provided in the table.

**Table 4:** Multiple linear regression analysis results with positive feelings subscale as dependent variable.

Dependent Variable: Positive Feelings	β+	SE++	*p*
Gender (Females vs. Males)	−1.45	1.05	0.173
Age	0.00	0.05	0.929
Educational level ^1^	−0.50	0.45	0.268
Married (yes vs. no)	−1.49	1.34	0.268
Have children (yes vs. no)	0.53	1.31	0.690
Living (with others vs. alone)	0.35	1.43	0.807
Health problems (yes vs. no)	0.13	0.95	0.888
Resilience score (CD-RISC)	0.29	0.03	<0.001
Total WPVB score	0.04	0.02	0.054

+ regression coefficient; ++ Standard Error; ^1^ greater values indicate greater educational level.

**Table 5:** Multiple linear regression analysis results with negative feelings subscale as dependent variable.

Predictor Variable	β	SE	*p*-Value	Significance
Gender (Females vs. Males)	−1.25	1.49	0.403	—
Age	−0.07	0.07	0.343	—
Educational level ^1^	−1.38	0.63	0.031	Significant − less education = more negative feelings
Married (yes vs. no)	−0.37	1.89	0.844	—
Have children (yes vs. no)	1.75	1.85	0.347	—
Living (with others vs. alone)	−1.59	2.01	0.431	—
Health problems (yes vs. no)	3.49	1.34	0.011	Significant − health issues = more negative feelings
Resilience score (CD-RISC)	−0.15	0.04	0.001	Highly significant − more resilience = fewer negative feelings
Total WPVB score	0.12	0.03	<0.001	Highly significant − more victimization = more negative feelings
Attack on personality	0.41	0.10	<0.001	Highly significant − strong impact
Attack on professional	0.32	0.09	0.001	Significant
Individual’s isolation from work	0.31	0.08	<0.001	Significant
Direct attack	0.28	0.19	0.137	—Not significant

^1^ Greater values indicate higher educational attainment. Note: WPVB scores were entered in turn due to intercorrelation between subscales.

## Data Availability

The data presented in this study are openly available in Aristotelis Koinis at [10.3934/PUBLICHEALTH.2019.1.79], reference number [65].

## Abbreviations

The following abbreviations are used in this manuscript:

CD-RISC	Connor–Davidson Resilience Scale
DYPE	Regional Health Authority
ED	Emergency Department
ICC	Intraclass Correlation Coefficient
SD	Standard Deviation
SE	Standard Error
SPSS	Statistical Package for the Social Sciences
WHO	World Health Organization
PANAS	Positive and Negative Affect Schedule
WPVB	Workplace Psychologically Violent Behaviors Questionnaire
IQR	Interquartile Range
CI	Confidence Interval
β	Beta Coefficient (used in regression analysis)
α	Cronbach’s Alpha (internal consistency reliability)
ρ (rho)	Spearman’s Rank Correlation Coefficient
r	Pearson’s Correlation Coefficient
*p*-value	Probability Value (used to assess statistical significance)

## Appendix A

**Table A1:** Intercorrelations of WPVB subscales.

	Attack on Personality	Attack on Professional	Individual’s Isolation from Work	Direct Attack	Total WPVB Score
Attack on personality	1.00	0.68 ***	0.61 ***	0.35 **	0.79 ***
Attack on professional		1.00	0.61 ***	0.56 ***	0.91 ***
Individual’s isolation from work			1.00	0.45 ***	0.80 ***
Direct attack				1.00	0.63 ***
Total WPVB score					1.00

** *p* < 0.01; *** *p* < 0.001.

## References

[1] Einarsen S., Hoel H., Zapf D., Cooper C.L. (2010). Bullying and Emotional Abuse in the Workplace: International Perspectives in Research and Practice.

[2] Johnson S.L., Rea R.E. (2009). Workplace bullying: Concerns for nurse leaders. J. Nurs. Adm..

[3] Giorgi G., Arcangeli G., Mucci N., Cupelli V. (2015). Economic stress in the workplace: The impact of fear of the crisis on mental health. Work.

[4] Watson D., Clark L.A., Tellegen A. (1988). Development and validation of brief measures of positive and negative affect: The PANAS scales. J. Pers. Soc. Psychol..

[5] Connor K.M., Davidson J.R. (2003). Development of a new resilience scale: The Connor-Davidson Resilience Scale (CD-RISC). Depress. Anxiety.

[6] Jackson D., Firtko A., Edenborough M. (2007). Personal resilience as a strategy for surviving and thriving in the face of workplace adversity: A literature review. J. Adv. Nurs..

[7] Hart P.L., Brannan J.D., De Chesnay M. (2014). Resilience in nurses: An integrative review. J. Nurs. Manag..

[8] Mealer M., Jones J., Newman J., McFann K.K., Rothbaum B., Moss M. (2012). The presence of resilience is associated with a healthier psychological profile in intensive care unit (ICU) nurses: Results of a national survey. Int. J. Nurs. Stud..

[9] Preacher K.J., Hayes A.F. (2004). SPSS and SAS procedures for estimating indirect effects in simple mediation models. Behav. Res. Methods Instrum. Comput..

[10] Hobfoll S.E., Folkman S. (2011). Conservation of resources theory: Its implication for stress, health, and resilience. The Oxford Handbook of Stress, Health, and Coping.

[11] Leymann H. (1996). The content and development of mobbing at work. Eur. J. Work Organ. Psychol..

[12] Adib S.M., Al-Shatti A.K., Kamal S., El-Gerges N., Al-Raqem M. (2002). Violence against nurses in healthcare facilities in Kuwait. Int. J. Nurs. Stud..

[13] Adriaenssens J., De Gucht V., Maes S. (2015). Causes and consequences of occupational stress in emergency nurses: A longitudinal study. J. Nurs. Manag..

[14] McGarry S., Girdler S., McDonald A., Valentine J., Wood F. (2013). Paediatric nurses’ work-related stress and supportive factors: A scoping review. J. Child Health Care.

[15] Healy S., Tyrrell M. (2011). Stress in emergency departments: Experiences of nurses and doctors. Emerg. Nurse.

[16] Purpora C., Blegen M.A. (2012). Horizontal violence and the quality and safety of patient care: A conceptual model. Nurs. Res. Pract..

[17] Johnson S.L. (2009). International perspectives on workplace bullying among nurses: A review. Int. Nurs. Rev..

[18] Wright B.E., Khatri N. (2015). Bullying among nurses and its relationship with organizational culture and leadership: A review of the literature. Int. J. Hum. Resour. Stud..

[19] Smith S.A. (2014). Mindfulness-based stress reduction: An intervention to enhance the effectiveness of nurses’ coping with work-related stress. Int. J. Nurs. Knowl..

[20] Delany C., Miller K.J., El-Ansary D., Remedios L., Hosseini A., McLeod S. (2015). Replacing stressful challenges with positive coping strategies: A resilience program for clinical placement learning. Adv. Health Sci. Educ. Theory Pract..

[21] Simons S.R., Mawn B. (2010). Bullying in the workplace—A qualitative study of newly licensed registered nurses. AAOHN J..

[22] Waschgler K., Ruiz-Hernández J.A., Llor-Esteban B., Jiménez-Barbero J.A. (2013). Vertical and lateral workplace bullying in nursing: Development of the Health Workplace Scale. J. Interpers. Violence.

[23] Nielsen M.B., Einarsen S. (2012). Outcomes of exposure to workplace bullying: A meta-analytic review. Work Stress.

[24] Giorgi G., Mancuso S., Fiz Perez F., Mucci N., Cupelli V., Arcangeli G. (2016). Bullying among nurses and its relationship with burnout and organizational climate. Int. J. Nurs. Pract..

[25] Kisa S., Dziegielewski S.F. (2009). Employee bullying in the workplace: A study of Turkish workers. J. Health Soc. Policy.

[26] Lin W.-Q., Wu J., Yuan L.-X., Zhang S.-C., Jing M.-J., Zhang H.-S., Luo J.-L., Lei Y.-X., Wang P.-X. (2015). Workplace Violence and Job Performance among Community Healthcare Workers in China: The Mediator Role of Quality of Life. Int. J. Environ. Res. Public Health.

[27] Soriano-Vázquez I., Cajachagua Castro M., Morales-García W.C. (2023). Emotional intelligence as a predictor of job satisfaction: The mediating role of conflict management in nurses. Front. Public Health.

[28] Demir D., Rodwell J. (2012). Psychosocial antecedents and consequences of workplace aggression for hospital nurses. J. Nurs. Scholarsh..

[29] Karatuna I., Gök S., Bozbay G. (2020). Relationship between mobbing exposure and emotional exhaustion: The mediating role of psychological resilience. Work.

[30] Aristidou L., Mpouzika M.D., Karanikola M.N. (2019). Exploration of Workplace Bullying in Emergency and Critical Care Nurses in Cyprus. Connect World Crit. Care Nurs..

[31] Lee J., Lee B. (2022). Psychological Workplace Violence and Health Outcomes in South Korean Nurses. Workplace Health Saf..

[32] Lin C.C., Liang H.F., Han C.Y., Chen L.C., Hsieh C.L. (2019). Professional resilience among nurses working in an overcrowded emergency department in Taiwan. Int. Emerg. Nurs..

[33] Mealer M., Jones J., Moss M. (2012). A qualitative study of resilience and posttraumatic stress disorder in United States ICU nurses. Intensive Care Med..

[34] Tawfik D.S., Profit J., Morgenthaler T.I., Satele D.V., Sinsky C.A., Dyrbye L.N., Tutty M.A., West C.P., Shanafelt T.D. (2018). Physician burnout, well-being, and work unit safety grades in relationship to reported medical errors. Mayo Clin. Proc..

[35] Laschinger H.K., Grau A.L. (2012). The influence of personal dispositional factors and organizational resources on workplace violence, burnout, and health outcomes in new graduate nurses: A cross-sectional study. Int. J. Nurs. Stud..

[36] Hsieh Y.L., Huang C.T., Tseng Y.H. (2021). How Emergency Nurses Develop Resilience in the Context of Workplace Violence: A Grounded Theory Study. J. Nurs. Scholarsh..

[37] Suldo S.M., Shaffer E.J., Riley K.N. (2008). A social-cognitive-behavioral model of academic predictors of adolescents’ life satisfaction. Sch. Psychol. Q..

[38] Yu F., Raphael D., Mackay L., Smith M., King A. (2019). Personal and work-related factors associated with nurse resilience: A systematic review. Int. J. Nurs. Stud..

[39] Hayes A.F. (2017). Introduction to Mediation, Moderation, and Conditional Process Analysis: A Regression-Based Approach.

[40] Preacher K.J., Hayes A.F. (2008). Asymptotic and resampling strategies for assessing and comparing indirect effects in multiple mediator models. Behav. Res. Methods.

[41] Hsieh H.F., Wang H.H., Ma S.C., Chang H.Y. (2021). The mediating effect of resilience on the relationship between workplace bullying and work-related outcomes among nurses in Taiwan. Int. J. Occup. Med. Environ. Health.

[42] Arrogante O., Aparicio-Zaldivar E. (2017). Burnout and Health among Critical Care Professionals: The Mediational Role of Resilience. Intensive Crit. Care Nurs..

[43] Tugade M.M., Fredrickson B.L. (2004). Resilient individuals use positive emotions to bounce back from negative emotional experiences. J. Pers. Soc. Psychol..

[44] Yıldırım A. (2019). The impact of workplace bullying on nurses’ psychological well-being and the mediating role of psychological resilience. J. Psychiatr. Nurs..

[45] Hegney D.G., Rees C.S., Eley R., Osseiran-Moisson R., Francis K. (2015). The contribution of individual psychological resilience in determining the professional quality of life of Australian nurses. Front. Psychol..

[46] Clark P., Crawford T.N., Hulse B., Polivka B.J. (2021). Resilience, moral distress, and workplace engagement in emergency department nurses. Res. Nurs. Health.

[47] Şimşek K. (2022). Investigation of the effect of secondary traumatic stress on psychological resilience in emergency nurses: A systematic review. J. Clin. Nurs..

[48] Jiang J., Liu S., Chi C., Liu Y., Xu J., Zeng L., Peng H. (2024). Experiences of compassion fatigue among Generation Z nurses in the emergency department: A qualitative study in Shanghai, China. BMC Nurs..

[49] Li J.-N., Jiang X.-M., Zheng Q.-X., Lin F., Chen X.-Q., Pan Y.-Q., Zhu Y., Liu R.-L., Huang L. (2023). Mediating effect of resilience between social support and compassion fatigue among intern nursing and midwifery students during COVID-19: A cross-sectional study. BMC Nurs..

[50] Kang H., Han K. (2021). Moderating effects of structural empowerment and resilience in the relationship between nurses’ workplace bullying and work outcomes: A cross-sectional correlational study. Int. J. Env. Res. Public Health.

[51] Fletcher D., Sarkar M. (2013). Psychological resilience: A review and critique of definitions, concepts, and theory. Eur. Psychol..

[52] Levin K.A. (2006). Study design III: Cross-sectional studies. Evid. Based Dent..

[53] Wang X., Cheng Z. (2020). Cross-sectional studies: Strengths, weaknesses, and recommendations. Chest.

[54] Setia M.S. (2016). Methodology series module 3: Cross-sectional studies. Indian J. Dermatol..

[55] McHugh M.D., Kutney-Lee A., Cimiotti J.P., Sloane D.M., Aiken L.H. (2011). Nurses’ widespread job dissatisfaction, burnout, and frustration with health benefits signal problems for patient care. Health Aff..

[56] Duffield C., Diers D., O’Brien-Pallas L., Aisbett C., Roche M., King M., Aisbett K. (2011). Nursing staffing, nursing workload, the work environment and patient outcomes. Appl. Nurs. Res..

[57] Polit D.F., Beck C.T. (2021). Nursing Research: Generating and Assessing Evidence for Nursing Practice.

[58] Laschinger H.K.S., Wong C.A., Cummings G.G., Grau A.L. (2010). Resonant leadership and workplace empowerment: The value of positive organizational cultures in reducing workplace incivility. Nurs. Econ..

[59] Einarsen S.V., Hoel H., Zapf D., Cooper C.L. (2020). Bullying and Harassment in the Workplace: Theory, Research and Practice.

[60] Shadish W.R., Cook T.D., Campbell D.T. (2002). Experimental and Quasi-Experimental Designs for Generalized Causal Inference.

[61] Zhang Y., Punnett L., Nannini A. (2016). Work environment and nursing staff well-being: An integrative review. Work.

[62] Bowling A. (2005). Mode of questionnaire administration can have serious effects on data quality. J. Public Health.

[63] Yildirim A., Yildirim D. (2007). Development and psychometric evaluation of workplace psychologically violent behaviours instrument. J. Clin. Nurs..

[64] Koinis A., Velonakis E., Tzavara C., Tzavella F., Tziaferi S. (2019). Psychometric properties of the workplace psychologically violent behaviors-WPVB instrument. Translation and validation in Greek health Professionals. AIMS Public Health.

[65] Beaton D.E., Bombardier C., Guillemin F., Ferraz M.B. (2000). Guidelines for the process of cross-cultural adaptation of self-report measures. Spine.

[66] Sousa V.D., Rojjanasrirat W. (2011). Translation, adaptation and validation of instruments or scales for use in cross-cultural health care research: A clear and user-friendly guideline. J. Eval. Clin. Pract..

[67] World Medical Association (2013). World Medical Association Declaration of Helsinki: Ethical Principles for Medical Research Involving Human Subjects. JAMA.

[68] Mukaka M.A. (2012). Guide to appropriate use of correlation coefficient in medical research. Malawi Med. J..

[69] Hayes A.F. (2013). Introduction to Mediation, Moderation, and Conditional Process Analysis: A Regression-Based Approach.

[70] Hayes A.F. (2009). Beyond baron and Kenny: Statistical mediation analysis in the new millennium. Commun. Monogr..

[71] Baron R.M., Kenny D.A. (1986). The moderator-mediator variable distinction in social psychological research: Conceptual, strategic, and statistical considerations. J. Pers. Soc. Psychol..

[72] Sobel M.E. (1982). Asymptotic confidence intervals for indirect effects in structural equation models. Sociol. Methodol..

[73] Laschinger H.K., Wong C.A., Grau A.L. (2012). The influence of authentic leadership on newly graduated nurses’ experiences of workplace bullying, burnout and retention outcomes: A cross-sectional study. Int. J. Nurs. Stud..

[74] Cai J., Wu S., Wang H., Zhao X., Ying Y., Zhang Y., Tang Z. (2023). The effectiveness of a workplace violence prevention strategy based on situational prevention theory for nurses in managing violent situations: A quasi-experimental study. BMC Health Serv Res..

[75] Kunzler A.M., Helmreich I., König J., Chmitorz A., Wessa M., Lieb K. (2020). Psychological interventions to foster resilience in healthcare professionals. Psychol. Med..

[76] Einarsen S., Hoel H., Cooper C. (2002). Bullying and Emotional Abuse in the Workplace: International Perspectives in Research and Practice.

[77] Fredrickson B.L. (2004). The broaden-and-build theory of positive emotions. Philos. Trans. R. Soc. Lond. B Biol. Sci..

[78] Bakker A.B., Oerlemans W.G., Cameron K.S., Spreitzer G.M. (2012). Subjective well-being in organizations. The Oxford Handbook of Positive Organizational Scholarship.

[79] McDonald G., Jackson D., Wilkes L., Vickers M.H. (2012). Personal resilience in nurses and midwives: Effects of a work-based educational intervention. Contemp. Nurse.

[80] Liao L., Guo N., Han Q., Qian Y., Xi H., Wang L. (2025). Long-Term Effect of a Comprehensive Active Resilience Education (CARE) Program for Increasing Resilience in Emergency Nurses Exposed to Workplace Violence: A Secondary Analysis of a 12-Week Follow-up Study. Int. Nurs. Rev..

[81] Vessey J.A., Demarco R., DiFazio R. (2010). Bullying, harassment, and horizontal violence in the nursing workforce: The state of the science. Annu. Rev. Nurs. Res..

[82] Javaheri A., Bartram T., Leggat S.G. (2021). Bullying in the emergency department: Associations with resilience and negative affect. Int. J. Env. Res. Public Health.

[83] Edwards D., Burnard P., Coyle D., Fothergill A., Hannigan B. (2000). Stress and burnout in community mental health nursing: A review of the literature. J. Psychiatr. Ment. Health Nurs..

[84] McCallin A. (2001). Interdisciplinary practice—A matter of teamwork: An integrated literature review. J. Clin. Nurs..

[85] Bailey T., Dollard M.F., Richards P.J. (2015). A National Standard for Psychosocial Safety Climate (PSC): PSC 41 as the Benchmark for Low Risk of Job Strain and Depressive Symptoms. Occup. Health Psychol..

[86] Leiter M.P., Laschinger H.K., Day A., Oore D.G. (2011). The impact of civility interventions on employee social behavior, distress, and attitudes. J. Appl. Psychol..

[87] McDonald G., Jackson D., Wilkes L., Vickers M.H. (2015). A work-based mindfulness intervention for nurses: A pilot study. J. Clin. Nurs..

[88] Smith B.W., Dalen J., Wiggins K., Tooley E., Christopher P., Bernard J. (2008). The brief resilience scale: Assessing the ability to bounce back. Int. J. Behav. Med..

[89] Einarsen S., Hoel H., Zapf D., Cooper C.L. (2011). Bullying and Harassment in the Workplace: Developments in Theory, Research, and Practice.

[90] Laschinger H.K., Leiter M.P., Day A., Gilin D. (2009). Workplace empowerment, incivility, and burnout: Impact on staff nurse recruitment and retention outcomes. J. Nurs. Manag..

[91] Etikan I., Musa S.A., Alkassim R.S. (2016). Comparison of convenience sampling and purposive sampling. Am. J. Theor. Appl. Stat..

